# Intermediate-risk pulmonary embolism: echocardiography predictors of clinical deterioration

**DOI:** 10.1186/s13054-022-04030-z

**Published:** 2022-06-04

**Authors:** Anthony J. Weekes, Denise N. Fraga, Vitaliy Belyshev, William Bost, Christopher A. Gardner, Nathaniel S. O’Connell

**Affiliations:** 1 Department of Emergency Medicine, Atrium Health’s Carolinas Medical Center, Charlotte, NC USA; 2 Department of Biostatistics and Data Science, Wake Forest School of Medicine, Winston-Salem, NC USA; 3 Present Address: Memorial Regional Medical Center, Mechanicsville, VA USA; 4 Present Address: Mid-Atlantic Emergency Medical Associates, Charlotte, NC USA

**Keywords:** Echocardiography, Pulmonary embolism, Right ventricle, Prognosis, Outcomes, Risk stratification

## Abstract

**Background:**

We determine the predictive value of transthoracic echocardiographic (TTE) metrics for clinical deterioration within 5 days in adults with intermediate-risk pulmonary embolism (PE).

**Methods:**

This was a prospective observational study of intermediate-risk PE patients. To determine associations of TTE and clinical predictors with clinical deterioration, we used univariable analysis, Youden’s index for optimal thresholds, and multivariable analyses to report odds ratios (ORs) or area under the curve (AUC).

**Results:**

Of 306 intermediate-risk PE patients, 115 (37.6%) experienced clinical deterioration. PE patients who had clinical deterioration within 5 days had greater baseline right ventricle (RV) dilatation and worse systolic function than the group without clinical deterioration as indicated by the following: RV basal diameter 4.46 ± 0.77 versus 4.20 ± 0.77 cm; RV/LV basal width ratio 1.14 ± 0.29 versus 1.02 ± 0.24; tricuspid annular plane systolic excursion (TAPSE) 1.56 ± 0.55 versus 1.80 ± 0.52 cm; and RV systolic excursion velocity 10.40 ± 3.58 versus 12.1 ± 12.5 cm/s, respectively. Optimal thresholds for predicting clinical deterioration were: RV basal width 3.9 cm (OR 2.85 [1.64, 4.97]), RV-to-left ventricle (RV/LV) ratio 1.08 (OR 3.32 [2.07, 5.33]), TAPSE 1.98 cm (OR 3.3 [2.06, 5.3]), systolic excursion velocity 10.10 cm/s (OR 2.85 [1.75, 4.63]), and natriuretic peptide 190 pg/mL (OR 2.89 [1.81, 4.62]). Significant independent predictors were: transient hypotension 6.1 (2.2, 18.9), highest heart rate 1.02 (1.00, 1.03), highest respiratory rate 1.02 (1.00, 1.04), and RV/LV ratio 1.29 (1.14, 1.47). By logistic regression and random forest analyses, AUCs were 0.80 (0.73, 0.87) and 0.78 (0.70, 0.85), respectively.

**Conclusions:**

Basal RV, RV/LV ratio, and RV systolic function measurements were significantly different between intermediate-risk PE patients grouped by subsequent clinical deterioration.

**Supplementary Information:**

The online version contains supplementary material available at 10.1186/s13054-022-04030-z.

## Introduction

Acute pulmonary embolism (PE) can cause an abnormal right ventricle (RV) to develop, which may cause clinical deterioration. Because there are no consistent definitions or assessments of abnormal RV (abnlRV), reports on abnlRV associations with clinical outcomes and prognostic performance vary [1–11]. With PE-provoked disruptions, RV dilatation precedes abnormal RV systolic function. Serum cardiac biomarkers, such as brain natriuretic peptide (BNP) and troponin, are commonly used as surrogates of myocardial dilatation and injury; however, neither provides definitive evidence of acute RV dilatation or RV injury. Although chest computed tomography (CT) provides information on abnlRV, reports are mixed on its diagnostic and prognostic accuracy compared to transthoracic echocardiography (TTE) [2, 4, 10, 12–15]. Although TTE can qualitatively and quantitatively assess RV size and systolic function, there is no consensus on which of many different TTE metrics define the standard for establishing the presence of clinically impactful RV abnormality [1, 11]. In addition, it is unlikely the different laboratory and image assessments or definitions of RV abnormality can be used interchangeably.

Although the American Society of Echocardiography (ASE) provides guidelines for RV chamber size and systolic function measurements for adults without cardiac or pulmonary disease, there are no disease-specific reference ranges for RV to grade disease severity [16–18]. The heterogeneity of abnlRV definitions in PE may impact study inclusion criteria, decisions for escalated intervention, and the components of prognostic models [3, 5, 6, 19, 20]. Physicians using different risk stratification models for determining abnlRV may arrive at different disposition decisions, clinical management considerations, and intervention decisions on any given PE patient. Meta-analyses, systematic reviews, and expert panels have identified this heterogeneity to be a problem [1–3, 8, 11]. A report from a large multinational registry shows that TTE findings of RV hypokinesis, enlarged right atrium, and intracardiac thrombus (rare) were associated with increased risk for 30-day mortality, but RV size and pressure variables were not studied and TTE was performed within 3 days [21]. RV size or systolic function is included as dichotomous predictors in some PE triaging strategies (e.g., Bova score and European Society of Cardiology guidelines); however, those strategies do not link the severity of RV abnormalities with their associated probability of immediate clinical deterioration [3, 10, 20, 22]. Early risk stratification remains important as the options for escalated PE intervention increase and definitions of therapeutic efficacy need clarification [5, 6, 9, 23–26].

Our primary goal was to identify TTE metrics that distinguish intermediate-risk PE patients at risk for clinical deterioration from those who do not experience death or clinical deterioration within 5 days. Our secondary goal was to compare the prognostic value of different TTE metrics with clinical predictors. Potential benefits of this pilot study include future development of prognostic models with a larger database of intermediate-risk PE patients to determine clinical usefulness.

## Methods

### Study design and setting

This was a prospective observational study within an integrated healthcare system that uses a multidisciplinary approach to the identification of intermediate- and high-risk PE patients, which triggers “Code PE” notifications to clinicians on duty. The research was approved by the institutional review board with a waiver of written informed consent. Study sites included eight Atrium Health emergency departments (EDs) in North Carolina (NC).

### Selection of participants

We used a prospective cohort design to identify and study adult patients (≥ 18 years) presenting to a participating ED, who had: (1) acute symptomatic PE as the primary ED diagnosis (by positive CT, high-probability ventilation/perfusion nuclear imaging, or point-of-care TTE findings highly suspicious of PE [e.g., RV dilatation on point-of-care, DVT findings]), (2) intermediate-risk PE classification, and (3) comprehensive TTE with RV-focused measurements completed within 24 h of PE diagnosis. PE risk stratification was based on review of vital signs, CT findings, natriuretic peptide, troponin, and bedside TTE findings at presentation. Patients classified as intermediate-risk PE were normotensive, with suspected or confirmed RV abnormalities. RV abnormalities were defined by either RV/LV ratio ≥ 1.0 on CT, abnormal cardiac biomarkers (either troponin elevation or BNP > 90 pg/mL), or abnormal point-of-care TTE (visual assessment of RV dilatation with or without RV systolic dysfunction or septal flattening or leftward bowing) [10].

Patients were excluded from this study if any of the following were true: (1) PE was not the primary diagnosis, (2) PE diagnosis was made > 12 h after admission, (3) PE was treated with anticoagulation before ED presentation, (4) recurrent PE after previous enrollment, (5) chronic PE resolving or unchanged in comparison with previous CT if available, (6) presence of an unstable rhythm at presentation or between measurements, (7) ongoing resuscitation or escalated PE interventions before TTE measurements, and (8) limited or no RV-focused measurements performed.

To create a quasi-control group, we randomly selected 25 PE patients from a previously reported PE registry (clinicaltrials.gov NCT03915925) [15], who had comprehensive TTE performed at Atrium Health and were not classified as intermediate or high risk.

Research coordinators at the main study site [Atrium Health’s Carolinas Medical Center] maintained logs of CTs ordered to follow up on PE-positive results. In addition, electronic order entry power plans with orders for comprehensive TTE were initiated for patients categorized at presentation as intermediate- and high-risk PE.

### Data collection and processing

We predefined variables during pre-enrollment meetings and specified locations of data within source documents in the EMR. In addition, our institutional informatics and analytics department provided reports on Code PE activations, including automated electronic medical record (EMR) data extraction for demographics and initial and worst vital signs within the first 3 h of ED stays. Examples include transient hypotension (systolic blood pressure < 90 mmHg for less than 15 min), highest heart rate (beats per minute), highest respiratory rate (breaths per minute), and lowest systolic blood pressure (mmHg) within 3 h of presentation. Lowest O_2_ saturation is the lowest oxygen saturation on room air oxygen within 3 h of initial vital signs. Manual data extraction from EMR was performed for PE risk factors, comorbidities, and clinical outcomes by trained study staff. Study data were entered into a standard electronic form managed within Research Electronic Data Capture (REDCap) tools hosted at Atrium Health’s Carolinas Medical Center (Charlotte, NC).

### Measurements

#### Laboratory measurements

Laboratory cardiac biomarker testing was obtained for patients with dyspnea or chest pain as per usual medical care or upon confirmation of PE during ED evaluation. We used a troponin i-STAT cardiac troponin test cartridge (Abbott Point of Care, Abbott Park, IL) measured in ng/mL for troponin I or high-sensitivity troponin assay. We used the i-STAT BNP test cartridge (Abbott Point of Care, Abbott Park, IL) measured in pg/mL. We evaluated laboratory values in two ways: continuous and binary. For the binary evaluations, normal point-of-care BNP measurements were considered to be below 90 ng/mL. Normal values for troponin I were less than 0.07 ng/mL. Normal values for high-sensitivity troponin were less than 12 for females and less than 20 for males.

#### Echocardiography measurements

Cardiac sonographers, certified by the American Registry of Diagnostic Medical Sonographers, performed all comprehensive TTE within the institution's echocardiography laboratory accredited by Intersocietal Commission for the Accreditation of Echocardiography Laboratories. Trained sonographers followed a protocol and standards established and operationalized by the Code PE scientific advisory committee, cardiology leaders, and echocardiography managers of the participating hospitals [16, 27]. The RV-focused view was used to measure RV end-diastolic internal diameter at the base, mid-section, and at its major axis (RV base to apex or length). LV basal end-diastolic measurements were performed in the parasternal long axis. RV systolic function measurements of tricuspid annular plane systolic excursion (TAPSE) and RV annulus peak systolic velocity (S’) were performed using M mode at the RV free wall annulus and tissue Doppler of the basal segment, respectively. RV systolic pressure (RVSP) was determined by tricuspid regurgitation peak velocity using continuous Doppler. RV/LV basal diameter ratio (RV/LV) was calculated. TTE images were saved as digital files, wirelessly transmitted, and archived in the secure local server and portal system, Merge Cardio™ (IBM Watson Health). Staff cardiologists interpreted TTE images and measurements and were blind to the study and patient clinical outcomes.

### Outcome measures

The primary composite outcome consisted of death, circulatory or respiratory deterioration (including cardiac arrest, respiratory failure, new unstable dysrhythmia, sustained hypotension treated with either fluid or adrenergic agents), or escalated PE intervention within 5 days of PE diagnosis. The secondary outcome extended the time frame to 30 days after PE and included all components of the primary outcome in addition to major bleeding episodes, recurrence of venous thromboembolism (VTE), and subsequent hospitalization. Detailed definitions of our previously reported clinical deterioration events and secondary outcomes are available as an appendix accompanying the online article [15, 28].

Study staff monitored enrolled patients during the hospitalization for clinical deterioration endpoints described above. The clinical status of patients who were discharged directly from the ED (or early discharge from the hospital) were ascertained by review of EMR for subsequent hospitalizations for reasons explicitly documented as related to PE. Finally, we monitored for major bleeding within 5 and 30 days of hospital admission. We used the International Society on Thrombosis and Hemostasis definition of major bleeding, which is defined as symptomatic bleeding in a critical organ area, bleeding causing a decrease in hemoglobin level of greater than 2 g/dL, or fatal bleeding [29].

### Primary data analysis

We used Peduzzi’s rule for logistic regression (outcome dichotomous yes/no) to justify our determination of the appropriate sample size [30]. This rule states that the maximum number of independent variables is no more than N/10, where N is the number of observations (subjects) in the smaller of the two groups (outcome yes or outcome no). According to this formula, 120 subjects were needed in the smaller subgroup (clinical deterioration = yes): 120 subjects/10 = 12 variables. Based on prior studies at our institution, we conservatively estimated a 40% occurrence of the primary outcome in our subjects with intermediate-risk PE or worse. Thus, we selected a sample size of 260 to detect a correlation of 0.2 between any of the TTE metrics with an alpha 0.05 and power of 0.9. We aimed to enroll 300 patients to exceed the desired sample size and allow for missing data and loss to follow-up, while providing robust analyses for our two study objectives.

In addition to TTE metrics, we evaluated patient demographics, comorbidities, and standard clinical factors (e.g., blood pressure, body mass index) as predictors. For summary statistics, continuous variables were described by their means with standard deviations or medians with interquartile ranges. Categorical variables were summarized by frequency counts and percentages. We used Student’s *t* tests for continuous data and chi-square tests for categorical data for univariable tests of each variable by our primary and secondary endpoints. All statistical analyses were two-tailed and conducted at alpha level of 0.05.

#### Multivariable analyses

To investigate the prognostic performance of TTE and laboratory measurements for clinical deterioration within days, we generated statistical inferences while assembling two tiers of prognostic models: (1) Youden’s index for optimal cutoff values, and (2) both logistic regression and random forest methods of prognostic model development.

As the first tier, for each continuous TTE variable and its respective univariable logistic regression model, we obtained an optimal cut-point for prognosis of 5-day clinical deterioration based on Youden’s index (maximizing sensitivity and specificity).

For the second tier, we used the process of prognostic model development and testing for clinical deterioration with multivariable logistic regression and random forest analyses. While the dataset available was not large enough for developing a prediction model ready for clinical use or implementation, we sought to assess the feasibility, performance, and utility such a model may provide in practice. We assessed two methods for prediction model development: logistic regression and random forest, using the “final” logistic model from the logistic regression analysis.

#### Logistic regression

To assess associations between the clinical predictors and outcomes, we performed a series of logistic regression (LR) analyses. For both primary and secondary outcomes, we first fit an “abnlRV only” model with clinical deterioration as a function of only our defined abnlRV predictors of interest. Given issues with multicollinearity due to high correlation among several abnlRV variables, we developed a “reduced abnlRV” model from the full abnlRV model using backward selection based on Akaike’s information criterion (AIC). Next, we defined our “full” model to include the significant abnlRV predictors from the reduced abnlRV model and all significant clinical (non-abnlRV) predictors based on univariable associations. From our “full” model, we developed our final logistic model via backward selection based on AIC. Statistical significance was defined by *p* < 0.05.

#### Random forest

First, we fit a random forest (RF) model using a full dataset (i.e., without splitting into test and training datasets) for both primary and secondary outcomes. We assessed variable importance plots based on mean decrease in accuracy to investigate the most important predictors of clinical deterioration and used “out-of-bag” prediction estimates to evaluate model fit in terms of F1 score, sensitivity, specificity, positive predictive value (PPV), and negative predictive value (NPV).

RF out-of-bag estimates have been shown to demonstrate consistent and unbiased results of model performance, without the requirement of needing separate test and training datasets [31]. This artifact of RFs is advantageous in our prediction model development given the limited dataset from which we were building. Next, we compared LR and RF prediction metrics. (Detailed methods are available in the Appendix accompanying the online article.)

#### Missing data

We used RF imputation to impute missing values across all predictors, except for tricuspid regurgitation jet velocity and RVSP, which when attempted or performed were classified as derived variables for trace or undetectable measurements [32, 33]. We conducted a sensitivity analysis by comparing prediction models based on imputed datasets with those based on complete case datasets.

## Results

### Characteristics of study subjects

**Fig. 1:**
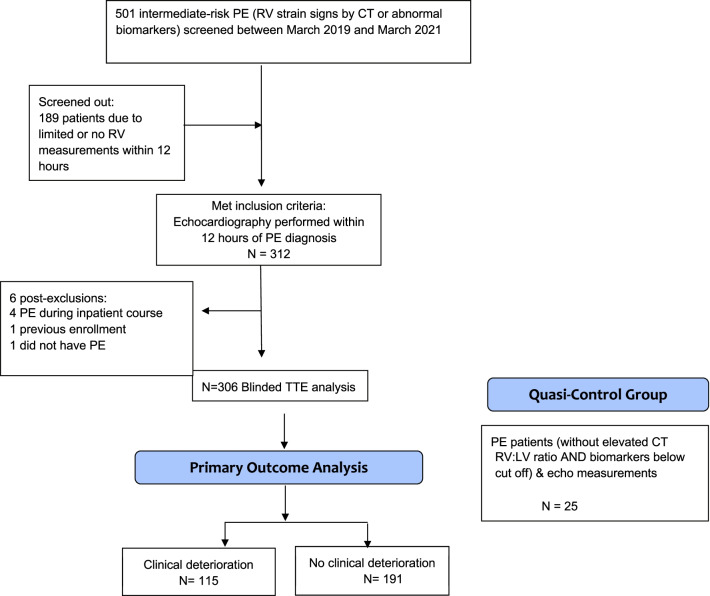
Screening and patient flow diagram. *Abbreviations*: *PE* pulmonary embolism, *RV* right ventricle, *TTE* transthoracic echocardiography, *CT RV/LV* computed tomography right ventricle-to-left ventricle basal diameter ratio

Between March 2019 and March 2021, we screened 501 Code PE patients; 306 intermediate-risk patients met criteria for complete analysis as shown in Fig. 1, 303 (98%) of whom had CT with RV/LV ratios reported. Of the remaining 3 patients without CT, two (0.6%) had high-probability ventilation/perfusion mismatch findings, and two had RV dilatation by point-of-care TTE (one with coexisting lower extremity DVT and one patient had clot in transit on point-of-care TTE as the confirmatory study). All 306 PE patients had troponin measurements and 191 (62.4%) had troponin elevations. Of the 306, 293 patients (95.7%) had BNP measurements, 192 of whom (65.5%) had BNP elevation. Of the 303 with CT, 237 (78.2%) had CT with RV/LV ratio ≥ 1.0.

Additional file 2: Table S1 shows clinical characteristics of the 306 intermediate-risk patients at ED presentation (mean age 60.5 [SD 16.2] years; 50.3% female). Sixty-two percent of patients identified as Caucasian, while 36% identified as African-American. Approximately 20% of patients had oxygen saturations ≤ 92%; 77 patients (25.1%) had shock index ≥ 1.0 at presentation; and 28 (9.2%) had systolic blood pressure ≤ 90 mmHg within 3 h of initial presentation. Twenty-six of the 306 patients (8.5%) were considered to have active bleeding or high risk of bleeding, including high-risk postoperative state. Anticoagulation was administered to 294 (96.1%) of 306 patients during the ED course. Of the 294 receiving anticoagulation, 194 (66%) received low molecular weight heparin, 101 (34.4%) received unfractionated heparin, and 1 patient (0.3%) was given oral factor Xa inhibitor in the ED.

Of the intermediate-risk PE patients without anticoagulation initiated *within the ED course*, seven were transported to inpatient units and received early thrombolysis, and one had anticoagulation delayed until head CT was completed before thrombolysis was administered. Two of the acute PE patients had active bleeding while already on anticoagulation (with therapeutic levels). The proportion with CT RV/LV ratio ≥ 1.0 was not significant between outcome groups.

Table 1 and Additional file 3: Table S2 show assessed RV variables overall and as stratified by primary outcome and secondary outcomes, respectively. Within 5 days, 115 (37.6%) patients experienced one or more clinical deterioration events or required an emergent in-hospital intervention. There were 3 (1.0%) deaths; 66 patients (21.6%) had one or more escalated PE interventions; 43 (14.1%) had symptomatic sustained hypotension addressed with intravenous fluid boluses; 31 (10.1%) had respiratory failure; 31 (10.1%) had new dysrhythmia requiring treatment; 20 (6.5%) had sustained hypotension treated with adrenergic agents; and 12 patients (3.3%) had cardiac arrest. There were no clinical deterioration events among the quasi-control group.

**Table 1:** Right ventricle assessment variables by primary outcome within 5 days*

	Clinical deterioration at 5 days	No clinical deterioration (CD)		Overall		*p* value comparing CD in cases (controls excluded)
	Case (*N* = 115)	Case (*N* = 191)	Control (*N* = 25)	Case (*N* = 306)	Control (*N* = 25)	
*Chamber dimensions*						
Right ventricle basal width (cm)						
Mean (SD)	4.46 (0.769)	4.20 (0.765)	3.81 (0.700)	4.30 (0.776)	3.81 (0.700)	
LV basal width (cm)						
Mean (SD)	4.07 (0.837)	4.25 (0.698)	4.54 (0.573)	4.18 (0.757)	4.54 (0.573)	0.0562
Missing	2 (1.7%)	4 (2.1%)	0 (0%)	6 (2.0%)	0 (0%)	
Right ventricle mid-width (cm)						
Mean (SD)	3.77 (0.763)	3.43 (0.906)	3.20 (0.819)	3.56 (0.870)	3.20 (0.819)	< 0.001
Missing	2 (1.7%)	2 (1.0%)	0 (0%)	4 (1.3%)	0 (0%)	
RV/LV basal width ratio						
Mean (SD)	1.14 (0.294)	1.02 (0.243)	0.844 (0.123)	1.06 (0.270)	0.844 (0.123)	< 0.001
Missing	2 (1.7%)	4 (2.1%)	0 (0%)	6 (2.0%)	0 (0%)	
*Systolic function*						
Tricuspid annular plane systolic excursion (TAPSE) (cm)						
Mean (SD)	1.56 (0.546)	1.80 (0.517)	2.06 (0.494)	1.71 (0.539)	2.06 (0.494)	< 0.001
Missing	7 (6.1%)	5 (2.6%)	0 (0%)	12 (3.9%)	0 (0%)	
RV free wall systolic excursion velocity S', cm/s						
Mean (SD)	10.4 (3.58)	12.1 (12.5)	14.0 (3.04)	11.4 (10.1)	14.0 (3.04)	0.101
Missing	9 (7.8%)	14 (7.3%)	1 (4.0%)	23 (7.5%)	1 (4.0%)	
Right-sided pressure Estimated PA pressure (mmHg)						
Mean (SD)	47.8 (14.7)	48.0 (19.0)	36.2 (13.4)	47.9 (17.4)	36.2 (13.4)	0.918
Missing	14 (12.2%)	37 (19.4%)	14 (56.0%)	51 (16.7%)	14 (56.0%)	
RVSP						
Missing or not detected	14.0 (12.2%)	37.0 (19.4%)	14.0 (56.0%)	51.0 (16.7%)	14.0 (56.0%)	0.093
Less than 35 mmHg (# of patients)	17.0 (14.8%)	43.0 (22.5%)	6.00 (24.0%)	60.0 (19.6%)	6.00 (24.0%)	
Between 35 and 47.5 mmHg	33.0 (28.7%)	42.0 (22.0%)	3.00 (12.0%)	75.0 (24.5%)	3.00 (12.0%)	
Between 47.5 and 56 mmHg	26.0 (22.6%)	29.0 (15.2%)	1.00 (4.0%)	55.0 (18.0%)	1.00 (4.0%)	
Greater than 56 mmHg	25.0 (21.7%)	40.0 (20.9%)	1.00 (4.0%)	65.0 (21.2%)	1.00 (4.0%)	
*Laboratory cardiac biomarkers*						
Initial BNP level (pg/mL)						
Mean (SD)	464 (683)	250 (357)	42.2 (54.8)	329 (512)	42.2 (54.8)	0.003
Missing	6 (5.2%)	6.00 (3.1%)	0 (0%)	12.0 (3.9%)	0 (0%)	
Initial troponin level (ng/mL)						
Mean (SD)	0.22 (0.329)	0.25 (0.77)	0.01 (0.014)	0.24 (0.635)	0.01 (0.0139)	0.69
Median [Min, Max]	0.09 [0, 1.38]	0.04 [0, 6.34]	0 [0, 0.04]	0.06 [0, 6.34]	0 [0, 0.0400]	
Missing	54 (47.0%)	98 (51.3%)	5 (20.0%)	152 (49.7%)	5 (20.0%)	
Initial high-sensitivity troponin, ng/L						
Mean (SD)	270 (448)	228 (1210)	11.1 (5.15)	243 (1000)	11.1 (5.15)	0.75
Median [Min, Max]	113 [5.00, 2670]	30.5 [0, 12600]	11.0 [6.00, 19.0]	49.0 [0, 12600]	11.0 [6.00, 19.0]	
Missing	53 (46.1%)	81 (42.4%)	14 (56.0%)	134 (43.8%)	14 (56.0%)	
Troponin elevation?						
Elevated	86.0 (74.8%)	105 (55.0%)	3 (12.0%)	191 (62.4%)	3.00 (12.0%)	< 0.001
Missing	0 (0%)	0 (0%)	1 (4.0%)	0 (0%)	1 (4.0%)	

^*^ The American Society of Echocardiography reported that the normal range values involve values within 2 standard deviations of the mean measurement. The following normal ranges of echocardiographic measurements were derived from patients without cardiac or pulmonary disease [16]:

RV basal diameter, 2.5–4.1 cm; RV mid-diameter, 1.9–3.5 cm; RV major length, 5.9–8.3 cm

LV diastolic basal diameter, male: 3.9–5.3 cm; RV/LV basal diameter ratio 0.6; LV ejection fraction 53–73%; tricuspid annular planar systolic excursion (TAPSE), 1.7–3.1 cm; S’: peak systolic velocity, 6.0–13.4 cm/s; and right ventricular systolic pressure (RVSP) 12–16 mmHg

*Abbreviations*: *CD* clinical deterioration, *abnlRV* abnormal right ventricle features, *RV* right ventricle, *LV* left ventricle, *PA* pulmonary artery, *RVSP* right ventricle systolic pressure, *BNP* natriuretic peptide

As shown in Table 1, intermediate-risk PE patients with clinical deterioration within 5 days had greater RV dilatation and worse systolic function than those without clinical deterioration as indicated by the following: RV basal diameter 4.46 ± 0.77 versus 4.20 ± 0.77 cm; RV/LV basal width ratio 1.14 ± 0.29 versus 1.02 ± 0.24; TAPSE 1.56 ± 0.55 versus 1.80 ± 0.52 cm; and RV systolic excursion velocity 10.4 ± 3.58 versus 12.1 ± 12.5 cm/s, respectively. In comparison with the intermediate-risk group, the quasi-control group of PE patients had less RV dilatation and better systolic function (mean RV basal diameter 3.8 ± 0.7 cm, RV/LV ratio 0.84 ± 0.12, TAPSE 2.06 ± 0.49 cm, systolic excursion velocity 14.0 ± 3.04 cm/s); their values were close to or within the normal range of values published by the ASE [11, 16–18, 34–36].

**Fig. 2:**
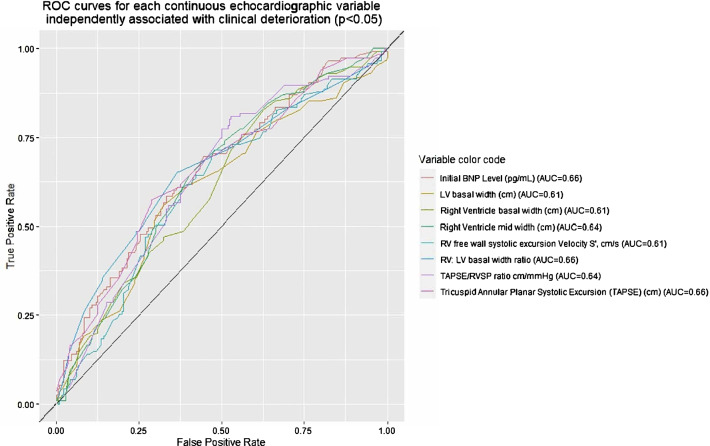
Receiver operating characteristic curves of optimal thresholds for predicting clinical deterioration. ROC curves for each continuous RVD variable independently associated with clinical deterioration (*p* < 0.05). *Abbreviations*: *ROC* receiver operating characteristic, *RVD* right ventricle abnormality, *BNP* brain natriuretic peptide, *AUC* area under the curve, *LV* left ventricle, *RV* right ventricle, *TAPSE* tricuspid annular planar systolic excursion, *RVSP* right ventricle systolic pressure

To address a trade-off of false positives and false negatives, we used Youden’s index to determine the optimal thresholds for predicting clinical deterioration. Optimal thresholds were: RV basal width 3.9 cm (OR 2.85 [1.64, 4.97]), RV-to-left ventricle (RV/LV) ratio 1.08 (OR 3.32 [2.07, 5.33]), TAPSE 1.98 cm (OR 3.3 [2.06, 5.3]), systolic excursion velocity 10.10 cm/s (OR 2.85 [1.75, 4.63]), and natriuretic peptide 190 pg/mL (OR 2.89 [1.81, 4.62]). However, the corresponding receiver operating characteristic (ROC) curves and AUC shown in Fig. 2 and Table 2 are modest. For example, based on our data, a TAPSE < 1.83 cm results in the optimal threshold for predicting higher risk of clinical deterioration based on TAPSE alone, such that patients with TAPSE < 1.83 had 3.35 times greater odds of clinical deterioration than those with TAPSE ≥ 1.83 cm. Using this threshold for prediction of clinical deterioration results in 57% sensitivity, 71% specificity, 52% PPV, and 76% NPV. Similar values for the other variables are given in Table 2.

**Table 2:** Optimal thresholds (determined by Youden’s index) with prognostic metrics

Variable	Optimal threshold	*p* value	Sensitivity	Specificity	PPV	NPV	AUC	OR
LV ejection fraction (%)	59	0.3	0.23 (0.16, 0.31)	0.86 (0.81, 0.9)	0.47 (0.34, 0.59)	0.68 (0.62, 0.73)	0.52 (0.45, 0.58)	1.83 (1.03, 3.25)
LV basal width (cm)	4.4	0.01	0.56 (0.47, 0.65)	0.68 (0.62, 0.74)	0.48 (0.40 0.57)	0.74 (0.68, 0.8)	0.61 (0.54, 0.67)	2.67 (1.68, 4.26)
Tricuspid annular plane systolic excursion (TAPSE) (cm)	1.98	0.00	0.57 (0.47, 0.66)	0.71 (0.66, 0.78)	0.52 (0.43, 0.6)	0.76 (0.7, 0.81)	0.66 (0.59, 0.72)	3.35 (2.06, 5.30)
Right ventricle mid-width (cm)	3.5	< 0.01	0.64 (0.56, 0.73)	0.6 (0.53, 0.66)	0.46 (0.38, 0.54)	0.76 (0.69, 0.82)	0.64 (0.58, 0.7)	2.68 (1.68, 4.28)
RV/LV basal width ratio	1.08	< 0.01	0.64 (0.56, 0.73)	0.6 (0.55, 0.66)	0.49 (0.41, 0.57)	0.78 (0.71, 0.84)	0.66 (0.59, 0.72)	3.32 (2.07, 5.33)
Initial BNP level (pg/mL)	190	< 0.01	0.59 (0.50, 0.68)	0.67 (0.60, 0.73)	0.49 (0.40, 0.57)	0.75 (0.69, 0.82)	0.66 (0.60, 0.72)	2.89 (1.81, 4.62)
RV free wall systolic excursion velocity S', cm/s	10.10	0.01	0.72 (0.64, 0.8)	0.52 (0.46, 0.59)	0.45 (0.37, 0.52)	0.78 (0.71, 0.85)	0.61 (0.55, 0.68)	2.85 (1.75, 4.63)
TAPSE/RVSP ratio cm/mmHg	0.05	< 0.01	0.79 (0.72, 0.87)	0.48 (0.41, 0.55)	0.45 (0.38, 0.52)	0.81 (0.74, 0.88)	0.65 (0.59, 0.71)	3.52 (2.09, 5.94)
Right ventricle basal width (cm)	3.9	< 0.01	0.83 (0.76, 0.9)	0.38 (0.31, 0.44)	0.41 (0.35, 0.48)	0.8 (0.72, 0.88)	0.61 (0.55, 0.68)	2.85 (1.64, 4.97)
Tricuspid regurg peak velocity, m/s	2.50	0.94	0.95 (0.91, 0.99)	0.1 (0.06, 0.14)	0.36 (0.31, 0.41)	0.79 (0.63, 0.94)	0.55 (0.49, 0.61)	2.06 (0.81, 5.24)
RV/major length (cm)	5.20	0.83	1 (1, 1)	0.09 (0.05, 0.13)	0.37 (0.32, 0.42)	1 (1, 1)	0.51 (0.45, 0.57)	0.83

*Abbreviations*: *PPV* positive predictive value, *NPV* negative predictive value, *AUC* area under the curve, *OR* odds ratio, *RV* right ventricle, *LV* left ventricle, *BNP* brain natriuretic peptide, *TAPSE* tricuspid annular plane systolic excursion, *RSVP* right ventricle systolic pressure

Table 3 shows significant differences in TTE measurements between those with (N = 66) and without (N = 240) reperfusion interventions within 5 days. At baseline, the group receiving reperfusion interventions had larger RV size (RV basal diastolic width 4.53 [0.807] versus 4.24 [0.757] cm and RV/LV ratio 1.16 [0.30] versus 1.04 [0.26]), lower TAPSE (1.52 [0.41] versus 1.76 [0.56] cm), and a greater proportion with higher RV pressure and troponin elevation than those without reperfusion interventions.

**Table 3:** Summary statistics and univariable analyses for right ventricle assessment predictors by escalated PE intervention*

	Reperfusion within 5 days	No reperfusion		Overall		*p* value comparing cases (controls excluded)
	Case (*N* = 66)	Case (*N* = 240)	Control (*N* = 25)	Case (*N* = 306)	Control (*N* = 25)	
Right ventricle basal width (cm)						
Mean (SD)	4.53 (0.807)	4.24 (0.757)	3.81 (0.700)	4.30 (0.776)	3.81 (0.700)	0.0106
LV basal width (cm)						
Mean (SD)	4.04 (0.740)	4.22 (0.759)	4.54 (0.573)	4.18 (0.757)	4.54 (0.573)	0.0913
Missing	2 (3.0%)	4 (1.7%)	0 (0%)	6 (2.0%)	0 (0%)	
Right ventricle mid-width (cm)						
Mean (SD)	3.86 (0.727)	3.48 (0.888)	3.20 (0.819)	3.56 (0.870)	3.20 (0.819)	< 0.001
Missing	1 (1.5%)	3 (1.3%)	0 (0%)	4 (1.3%)	0 (0%)	
RV/LV basal width ratio						
Mean (SD)	1.16 (0.296)	1.04 (0.257)	0.844 (0.123)	1.06 (0.270)	0.844 (0.123)	0.00252
Missing	2 (3.0%)	4 (1.7%)	0 (0%)	6 (2.0%)	0 (0%)	
Tricuspid annular plane systolic excursion (TAPSE) (cm)						
Mean (SD)	1.52 (0.414)	1.76 (0.557)	2.06 (0.494)	1.71 (0.539)	2.06 (0.494)	< 0.001
Missing	5 (7.6%)	7 (2.9%)	0 (0%)	12 (3.9%)	0 (0%)	
Estimated PA pressure (mmHg)						
Mean (SD)	50.3 (13.9)	47.2 (18.3)	36.2 (13.4)	47.9 (17.4)	36.2 (13.4)	0.174
Missing	6 (9.1%)	45 (18.8%)	14 (56.0%)	51 (16.7%)	14 (56.0%)	
RVSP						
Missing or not detected	6.00 (9.1%)	45.0 (18.8%)	14.0 (56.0%)	51.0 (16.7%)	14.0 (56.0%)	< 0.001
Less than 35 mmHg	3.00 (4.5%)	57.0 (23.8%)	6.00 (24.0%)	60.0 (19.6%)	6.00 (24.0%)	
Between 35 and 47.5 mmHg	25.0 (37.9%)	50.0 (20.8%)	3.00 (12.0%)	75.0 (24.5%)	3.00 (12.0%)	
Between 47.5 and 56 mmHg	18.0 (27.3%)	37.0 (15.4%)	1.00 (4.0%)	55.0 (18.0%)	1.00 (4.0%)	
Greater than 56 mmHg	14.0 (21.2%)	51.0 (21.3%)	1.00 (4.0%)	65.0 (21.2%)	1.00 (4.0%)	
RV free wall systolic excursion velocity S', cm/s						
Mean (SD)	10.4 (3.26)	11.7 (11.3)	14.0 (3.04)	11.4 (10.1)	14.0 (3.04)	0.133
Missing	6 (9.1%)	17 (7.1%)	1 (4.0%)	23 (7.5%)	1 (4.0%)	
Initial BNP level (pg/mL)						
Mean (SD)	441 (551)	299 (499)	42.2 (54.8)	329 (512)	42.2 (54.8)	0.0683
Missing	4 (6.1%)	8 (3.3%)	0 (0%)	12(3.9%)	0 (0%)	
Initial troponin level (ng/mL)						
Mean (SD)	0.227 (0.356)	0.239 (0.693)	0.0105 (0.0139)	0.237 (0.635)	0.0105 (0.0139)	0.887
Median [Min, Max]	0.0700 [0, 1.38]	0.0500 [0, 6.34]	0 [0, 0.0400]	0.0600 [0, 6.34]	0 [0, 0.0400]	
Missing	33(50.0%)	119 (49.6%)	5 (20.0%)	152 (49.7%)	5 (20.0%)	
Initial high-sensitivity troponin, ng/L						
Mean (SD)	181 (193)	260 (1130)	11.1 (5.15)	243 (1000)	11.1 (5.15)	0.439
Median [Min, Max]	102 [8.00, 767]	38.0 [0, 12600]	11.0 [6.00, 19.0]	49.0 [0, 12600]	11.0 [6.00, 19.0]	
Missing	29 (43.9%)	105 (43.8%)	14 (56.0%)	134 (43.8%)	14 (56.0%)	
Troponin elevation?						
Elevated	49 (74.2%)	142 (59.2%)	3 (12.0%)	191 (62.4%)	3 (12.0%)	0.0361
Missing	0 (0%)	0 (0%)	1 (4.0%)	0 (0%)	1 (4.0%)	

^*^Escalated PE intervention = Reperfusion therapy (defined as systemic thrombolysis, catheter-directed thrombolysis, mechanical thrombectomy, surgical embolectomy), or placement on extracorporeal membrane oxygenation circuit

*Abbreviations*: *BNP* brain natriuretic peptide, *LV* left ventricle, *PA* pulmonary artery, *RV* right ventricle, *RVSP* right ventricular systolic pressure

### Logistic regression

The significant abnlRV variables from the best fitting abnlRV model were troponin, BNP, tricuspid regurgitant peak velocity, RV/LV basal width ratio, and TAPSE. These abnlRV variables were included with all other patient factors, from which we developed our final, best fitting logistic regression model through backward selection as described in the Methods section. Table 4 shows the estimated odds ratios, 95% confidence intervals, and p values for our best fitting logistic regression model for 5-day clinical deterioration. Significant independent predictors were: transient hypotension 6.1 (2.2, 18.9), highest heart rate within the initial 3 h 1.02 (1.00, 1.03), highest respiratory rate within initial 3 h 1.02 (1.00, 1.04), and RV/LV ratio 1.29 (1.14, 1.47). [Given an OR of 1.02, a patient whose maximum heart rate was 121 beats per minute (bpm) had a 2% greater odds of clinical deterioration than a patient whose maximum heart rate was 120 bpm. For initial O_2_ saturation, we observed a significant OR of 0.94. Patients with lower O_2_ had higher odds of clinical deterioration. For every percentage point decrease in maximum O_2_ within 3 h, a patient's odds of clinical deterioration increased by 6%.]

**Table 4:** Final logistic model for 5-day clinical deterioration

	**Clinical Deterioration within 5 days**		
Predictors	Odds ratios	Confidence interval	*p* value
Initial BNP level (per 100 pg/mL)	1.10	1.03–1.19	0.008
Tricuspid regurg peak velocity, m/s	0.77	0.58–1.01	0.071
RV/LV basal width ratio (per 10% point increase in ratio)	1.29	1.14–1.47	< 0.001
Age	0.97	0.96–0.99	0.010
Highest heart rate (within 3 h)*	1.02	1.00–1.03	0.039
Highest respiratory rate (within 3 h)**	1.02	1.00–1.04	0.109
Lowest O_2_ saturation within 3 h***	0.94	0.89–0.99	0.033
SBP < 90 mmHg for < 15 min prior to enrollment?	6.18	2.24–18.92	0.001
Indwelling vascular catheter? (e.g., portacath)	0.19	0.03–0.81	0.047
Congestive heart failure	2.83	0.82–9.51	0.095
Anticoagulant initiated in ED?	0.08	0.00–0.54	0.029
Observations	331		
R2 Tjur	0.318		

*Abbreviations*: *BNP* brain natriuretic peptide, *RV/LV* right ventricle-to-left ventricle ratio, *SBP* systolic blood pressure, *ED* emergency department

^*^For each additional maximum heart rate in beats per minute or **respiratory rate in breaths per minute within the first 3 h, the ORs were 1.02, i.e., their odds of clinical deterioration increased by 2%

^***^For initial O2 sat, we see an OR of 0.94 or every percentage point decrease in max O2 within 3 h, a patient's odds of clinical deterioration increased by 6%

Among the abnlRV variables, BNP (*p* = 0.008) and RV/LV basal width ratio (*p* < 0.001) were statistically significantly related to increased odds of clinical deterioration, while tricuspid regurgitant peak velocity was marginally significant (*p* = 0.062). TAPSE and elevated troponin were not independent predictors and were dropped from the model. Additional files 4 and 5: Tables S3 and S4 show reduced and full LR models for 30-day outcomes, respectively.

### Random forest analysis

**Fig. 3:**
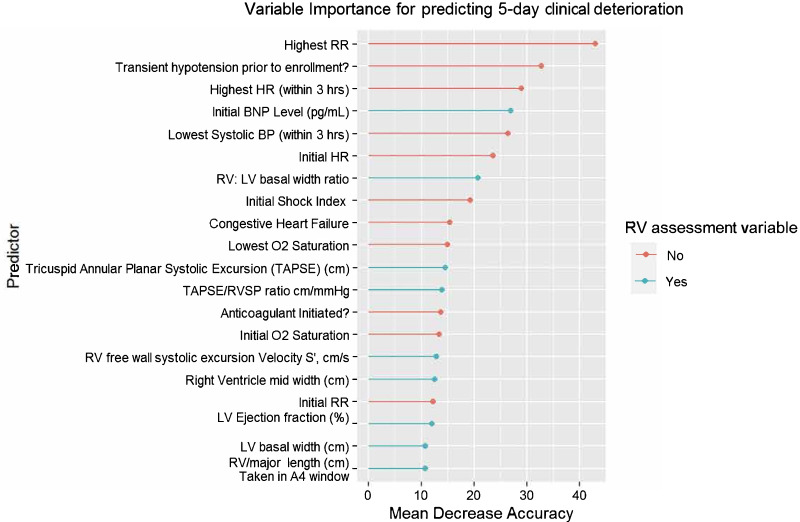
Variable importance plot for predicting 5-day clinical deterioration. *Abbreviations*: *A4* apical 4-chamber window, *BP* blood pressure, *LV* left ventricle, *RR* respiratory rate, *RV* right ventricle, *RVD* right ventricle abnormality, *RVSP* right ventricle systolic pressure, *TAPSE* tricuspid annular planar systolic excursion

We used out-of-bag error rates to estimate performance metrics and fit an RF prediction model for 5-day clinical deterioration (a binary outcome) and 30-day clinical deterioration. For both outcomes, we found multiple TTE metrics among the top 20 predictors (Fig. 3, Additional file 7: Fig. S1). Each of the selected components of our final LR model in the previous section was within the top 20 predictors in the RF model.

We achieved relatively good prediction metrics for both 5-day and 30-day clinical deterioration with correct prediction of 69% of clinical deterioration cases and 74% of non-clinical deterioration cases. Combined, this translates to a PPV of 59% and NPV of 82%. Comparing 5-day with 30-day prediction performance, there was similar sensitivity; however, the 5-day clinical deterioration model had slightly higher specificity and NPV, while the 30-day clinical deterioration predictive model had slightly higher PPV.

The full RF model performance metrics included AUC 0.78 (0.73, 0.83) for 5-day outcomes (Table 5). Calibration was not estimated from the full dataset RF models due to the inability to calculate predicted probabilities from out-of-bag estimates.

**Table 5:** Prognostic performance of full random forest model for primary and secondary outcomes (95% confidence intervals)

Outcome	F1 Score	Sensitivity	Specificity	Positive predictive value	Negative predictive value	Area under the curve (AUC)
5-day clinical deterioration	0.64 (0.59, 0.69)	0.70 (0.62, 0.79)	0.73 (0.67, 0.79)	0.58 (0.5, 0.66)	0.82 (0.77, 0.88)	0.78 (0.73, 0.83)
30-day clinical deterioration	0.63 (0.58, 0.68)	0.64 (0.57, 0.72)	0.68 (0.61, 0.74)	0.61 (0.53, 0.69)	0.71 (0.64, 0.77)	0.74 (0.68, 0.79)

### LR versus RF

In over 500 random data splits comparing LR and RF prediction models, both models performed about equally well in the test datasets. The mean AUC for prediction in the test dataset was 0.80 (95% confidence interval 0.73–0.87) and 0.78 (95% confidence interval 0.70–0.85) for the LR and RF models, respectively (Additional files 6: Table S5). Delong’s significance test of AUCs between the two models was not statistically significant for *any* of the 500 random test–validation data splits (mean *p* value = 0.66) [37]. Assuming classification thresholds of 0.50 for both models, the RF model was more sensitive on average (73% vs. 54%), with the trade-off of reduced specificity (69% vs. 87%). Similarly, the RF model demonstrated lower PPV (56% vs. 70%) and slightly higher NPV (83% vs. 78%) compared with the LR model. Given the clinical implications and negative consequences for failing to capture true cases of clinical deterioration, we investigated varying classification thresholds for predicting clinical deterioration. Thus, it may be beneficial to sacrifice specificity for better sensitivity. Investigating the prediction metrics in this context, we find that to achieve sensitivity of > 90%, we can use a classification threshold of 30%. Doing so yields a sensitivity of 91.6%, specificity of 45.1%, PPV of 42.2%, and NPV of 92.4%.

## Discussion

This prospective study investigated the ability to stratify several common TTE metrics in intermediate-risk PE patients by associating them with clinical deterioration or subsequent use of escalated PE interventions. In line with our primary goal, univariable analyses showed that intermediate-risk PE patients experiencing subsequent clinical deterioration had significantly greater RV chamber size and lower systolic function measurements compared to those not experiencing clinical deterioration. Overall, TTE metrics of RV dilatation and systolic dysfunction were worse in our intermediate-risk PE patients than in the quasi-control group. In turn, TTE metrics of the intermediate-risk group were outside the normal range of values published by the ASE, whereas those of the quasi-control group were close to or within the normal range [11, 16–18, 34–36]. Patients receiving reperfusion interventions had significantly larger RV diameter and lower (worse) RV systolic function metrics than patients not receiving reperfusion interventions.

Our secondary goal involved using multivariable analyses to compare the prognostic performance of TTE metrics with clinical predictors. We discovered clinical predictors (transient hypotension and highest heart rate and respiratory rate within 3 h) ranked higher as independent predictors than RV/LV ratio. Predicting clinical deterioration (risk stratification) remains important in this challenging group of PE patients because there are now more options for escalated PE intervention (other than full-dose thrombolysis) that do not compromise therapeutic efficacy or safety outcomes. The clinical implication is that more stringent risk classifications of severity of RV dilation and systolic function using TTE metrics are possible and may identify intermediate-risk PE patients at increased risk for clinical deterioration, who would benefit from increased consideration for escalated PE interventions.

We compared our findings with previous related reports. After using Youden’s index to acknowledge the trade-off of false positives and false negatives for subsequent clinical deterioration, we noted that our optimal thresholds for RV basal diameter (3.9 cm) and RV/LV ratio (1.02) were higher than thresholds used by Bova et al. to define abnormal RV in the Bova score (TTE RV/LV ratio > 0.9, RV diastolic diameter > 3.0 cm, RV dilatation, pressure > 30 mmHg) [20]. Compared to the report by Zanobetti et al. on TTE metrics in 120 consecutive PE patients at ED presentation [38], TTE metrics in our intermediate-risk PE patients had greater RV basal diameter (4.30 vs. 3.6 cm) and RV/LV ratio (1.07 vs. 0.75) and lower LV basal diameter (4.15 vs. 4.9 cm). For RV systolic function, previous reports found that TAPSE greater than 1.8–2.0 cm was associated with very low risk of deterioration or death, whereas TAPSE less than 1.5–1.6 cm was associated with 30-day mortality or need for escalated thrombolysis [39–41]. Our optimal cutoff for TAPSE (1.98 cm) was within the upper cutoff of normal ranges in previous reports focused on PE-related death at 30 days [40–43]. However, our study’s mean TAPSE measurement was less than 1.6 cm for those experiencing one or more clinical deterioration endpoints within 5 days and even worse (1.52 cm) for those with reperfusion interventions.

We observed increased RV/LV ratio and decreased LV basal diameter in our intermediate-risk PE cohort compared to the normal reference range, and significant measurement differences between those with clinical deterioration and those without [16, 17]. Decreased RV systolic function represents a more advanced pathophysiological stage of PE than having RV dilatation alone. RV dilatation and RV systolic dysfunction were determined to be distinct variables of importance.

Our report addresses recently identified high priority knowledge gaps and research opportunities by the 2018 American Thoracic Society Research Statement [8]. Few studies on acute pulmonary hypertension syndromes systematically compare TTE metrics to inform our decisions on prognosis or therapeutic efficacy [11]. Reports use TTE metrics for study inclusion, prognosis, and therapeutic efficacy, but criteria and definitions vary, at times with discordance [1–6, 9, 11, 23]. In some, the definitions involve qualitative assessments or unstated definitions of RV dilatation or abnormal RV systolic function [6, 19, 20, 44]. In many of the composite abnlRV studies, there was no rationale for the choice of metrics [11]. In some studies, there was discordance between TTE metrics [11]. Khemasuwan et al. studied the association of a wide array of TTE metrics with clinical outcomes in 211 critically ill PE patients in the intensive care unit and found RV/LV ratio, RVSP, and TAPSE were independently associated with hospital mortality [45]. In our study, RV/LV ratio and TAPSE were also found to be associated with clinical deterioration events within 5 days, but RVSP was not (p = 0.74). The implications for clinical research include recommendation that TTE metrics of RV size and systolic function should not be used interchangeably to define abnlRV either as inclusion criteria or as efficacy endpoints.

A report from the Pulmonary Embolism Response Team Consortium registry used four previously validated PE risk tools for 7-day mortality and generated modest AUCs between 0.62 and 0.67 [46]. For our study’s multivariable analyses, RF and LR prognostic models for 5-day clinical deterioration had similar mean AUC (0.78 and 0.80, respectively), which were not statistically different from Delong's test (*p* > 0.5) [37]. We recognize that eventual implementation of a clinically useful prognostic model will require more training data with independent validation sets. Our study method capitalized on the process of developing a prognostic model by contemporaneously stratifying TTE metrics and important clinical variables that determined statistical association with clinical outcomes of interest. Nevertheless, our results demonstrate that an accurate prediction model for predicting clinical deterioration is feasible and may provide clinically helpful patient care information. When applied to intermediate-risk PE, our results provided good evidence to support the continuation of this research using a larger dataset to develop a “final'' prediction model for clinical use in practice. The similar predictive performance between LR and RF is noteworthy because an equally accurate LR model would be more easily implemented in clinical practice than a machine learning RF model [47]. However, the prediction metrics of the RF model using the full dataset showed better performance than the average fit statistics of the LR model across 500 data splits.

The baseline model we have described uses a classification threshold of 0.5. (Predicted probability > 0.5 results in positive prediction.) False negatives may be costly and risky. Adjusting the “classification threshold” effectively allows one to tune the prediction model developed to a desired level of sensitivity and address overtriage and undertriage targets for clinical practice.

One of the strengths of our study is that we analyzed TTE metrics stratified by clinical outcomes. Our findings may be helpful in classifying the degree of abnormality (e.g., mild, moderate, severe) for measurements outside the normal range. These PE-focused TTE metrics can be obtained at bedside by emergency medicine or critical care physicians and may be considered (in combination with vital sign abnormalities, comorbidities, and elevated cardiac biomarkers) to further risk-stratify intermediate-risk PE and predict probability of subsequent clinical deterioration. Furthermore, these values may aid decision-making regarding preferred level of inpatient monitoring and considerations for emergent reperfusion if risk of clinical deterioration is felt to be high. Ultimately, more data are needed to further delineate acceptable cutoffs for these metrics in predicting deterioration.

Our study had several limitations, including the sample size, demographic limitations of a regional study, and unchallenged generalizability based on practice variation between physicians and facilities, the latter of which affected the decision to perform a reperfusion intervention (one component of our composite primary outcome). We predefined clinical deterioration endpoints to minimize variation in interpretation. We defined the endpoint of respiratory failure as the use of high-flow nasal cannula, bilevel positive airway pressure, or intubation, which would have the least disagreements among abstractors. It is also worth noting that not all TTE metrics were recorded for every enrolled patient. The LR odds ratio and coefficient results for RVSP go against conventional wisdom and experience. It is plausible that RV pressure does not linearly increase as PE severity worsens or that RVSP may have complex interactions with the onset, or progressive worsening, of RV systolic dysfunction. PE patients with missing values (not performed, technically difficult, or not possible) may have had qualitatively enlarged RV measurements that were clinically significant.

One of the independent predictors of clinical deterioration was not having initial anticoagulation administered in the ED. This was a statistical association without a causative association. The involved patients in our database were adequately treated. In these cases, the explanation for increased risk for primary outcome was not inadequate PE treatment. The lack of anticoagulation in the ED was due to recognition of the PE in the unique individual patients as being complex and high acuity. Those cases involved early decisions for escalated PE interventions or restraint due to concurrent bleeding or preexisting anticoagulation.

It is plausible that TTE cardiac output metrics, which we did not study, may have greater prognostic value and relevance than RV measurements for subsequent hemodynamic deterioration [48]. In addition, although we reported on single early TTE metrics, the direction and magnitude of acute changes may be important predictors of clinical deterioration [5, 7, 9, 23].

## Conclusions

In conclusion, intermediate-risk PE patients with 5-day clinical deterioration had significantly increased BNP and troponin, increased RV/LV ratio and RV basal diameter, and decreased LV basal diameter and RV systolic function (TAPSE and S’) measurements. TTE metrics were important in the RF prediction model, and the LR model had good prognostic performance. Although further work is needed to better understand the relationships between these variables and their independent prognostic ability, our results suggest that variables we identified (e.g., TAPSE, RV/LV basal width ratio, BNP) may provide simple, but useful criteria for assessing patient risk of 5-day clinical deterioration in intermediate-risk PE patients. Further development on a larger database and subsequent validation will allow optimization of lower and higher risk classification thresholds for prognostic modeling in intermediate-risk PE patients.

## Supplementary Information

**Additional file 1: Methods supplement.** Definitions of outcomes; ** Statistical methods.** Logistic regression model training and validation and comparison of models.

**Additional file 2: Table S1.** Summary statistics and univariable analyses for all predictor variables.

**Additional file 3: Table S2.** Echocardiography metrics by secondary outcome.

**Additional file 4: Table S3.** Reduced right ventricle assessment logistic regression model for 30-day clinical deterioration.

**Additional file 5: Table S4.** Final logistic model for 30-day clinical deterioration.

**Additional file 6: Table S5.** Comparison of random forest and logistic regression on 5-day clinical deterioration over 500 random test-validation data splits (mean metrics with 95% coverage intervals*)**.

**Additional file 7: Fig. S1.** Variable importance plot for random forest prognostic model for the secondary outcome. Abbreviations: A4 = apical 4-chamber window, BMI = body mass index, BP = blood pressure, CT = computed tomography, HR = heart rate, LV = left ventricle, Min = minimum, Max = maximum, RR = respiratory rate, RV = right ventricle, RVD = right ventricle abnormality, RVSP cat = right ventricle systolic pressure category, SD = standard deviation, TAPSE = tricuspid annular planar systolic excursion.

## Data Availability

The datasets used or analyzed during the current study are available from the corresponding author on reasonable request.

## Abbreviations

A4: Apical 4-chamber window
abnlRV: Abnormal right ventricle features
AIC: Akaike’s information criterion
ASE: American Society of Echocardiography
AUC: Area under the curve
BMI: Body mass index
BNP: Brain natriuretic peptide
BP: Blood pressure
CT: Computed tomography
DVT: Deep venous thrombosis
ED: Emergency department
EMR: Electronic medical record
HR: Heart rate
LR: Logistic regression
LV: Left ventricle
NC: North Carolina
NPV: Negative predictive value
PA: Pulmonary artery
PE: Pulmonary embolism
PPV: Positive predictive value
RF: Random forest
ROC: Receiver operating characteristic
RR: Respiratory rate
RV: Right ventricle
RVLV: Right ventricle-to-left ventricle basal diameter ratio
RVSP: Right ventricle systolic pressure
S’: Right ventricle free wall systolic excursion velocity
TAPSE: Tricuspid annular planar systolic excursion
TTE: Transthoracic echocardiography
VTE: Venous thromboembolism
