# Supplementary material for: Intermediate-risk pulmonary embolism: echocardiography predictors of clinical deterioration
Source: Crit Care. 2022 Jun 4;26:160. doi: 10.1186/s13054-022-04030-z (PMC9166499; doi:10.1186/s13054-022-04030-z)
Supplement: Supplementary file 1 — Additional file 1: Methods supplement. Definitions of outcomes; Statistical methods. Logistic regression model training and validation and comparison of models. [file 13054_2022_4030_MOESM1_ESM.pdf]

METHODS SUPPLEMENT

Definition of Outcomes:

Deaths were classified as PE-related when the site investigator reviewed the case and determined death was not likely to be due to another cause, such as septic shock or acute myocardial infarction. Circulatory or respiratory deterioration included any of the following: cardiac arrest, severe respiratory failure, symptomatic hypotension, and new onset dysrhythmia. Cardiac arrest was defined as the development of an unstable cardiac rhythm or absent electrical activity requiring cardiopulmonary resuscitation or advanced cardiac life support for asystole, pulseless electrical activity, ventricular fibrillation, or unstable ventricular tachycardia.

Severe respiratory failure was determined if the patient required emergent interventions with mechanical ventilation. Symptomatic sustained hypotension was defined as administration of 500 mL or more of intravenous fluids for volume expansion in response to hypotension. This was verified by electronic order entry documentation for intravenous fluid volumes of > 500 ml administered within 15 minutes. Hypotension requiring catecholamine treatment was determined by administration of norepinephrine, dopamine, or epinephrine infusion. New onset dysrhythmia was defined by the onset of atrial fibrillation with rapid ventricular response, atrial flutter, supraventricular tachycardia, stable ventricular tachycardia, or bradycardia that was not evident at ED presentation. Escalated PE intervention included reperfusion therapy (defined as systemic thrombolysis, catheter-directed thrombolysis, mechanical thrombectomy, surgical embolectomy), or placement on extracorporeal membrane oxygenation circuit.

Statistical methods: logistic regression model training and validation and comparison of models:

We assessed performance of our ‘final’ logistic model in terms of its prediction performance compared to an RF model. Unlike RF models, LR prediction models do not have an internal validation metric, and thus require a training and validation dataset. We used a 70:30 split for training and validation datasets. Using the predictors identified from the best fitting logistic model developed in the previous section, we fit an LR model to our training data and assessed performance in the test data using the following metrics: area under the curve (AUC), F1, sensitivity, specificity, PPV, and NPV. Using the same training and test datasets, we then fit an RF model, calculated the same prediction statistics, and compared the RF model to the LR model. We compared the models statistically using DeLong’s test for differences in AUC. Given the relatively small dataset for prediction model development, there was significant variability in prediction performance dependent on the variation in the random data split. To account for this, we repeated our data split and comparison of models 500 times, and compared mean model performance metrics, as well as summary statistics for the DeLong test p-values.
