# Supplementary material for: Intermediate-risk pulmonary embolism: echocardiography predictors of clinical deterioration
Source: Crit Care. 2022 Jun 4;26:160. doi: 10.1186/s13054-022-04030-z (PMC9166499; doi:10.1186/s13054-022-04030-z)
Supplement: Supplementary file 2 — Additional file 2: Table S1. Summary statistics and univariable analyses for all predictor variables. [file 13054_2022_4030_MOESM2_ESM.pdf]

Table S1: Summary statistics and univariable analyses for all predictor variables

	Clinical Deterioration at 5 Days	No Clinical Deterioration (CD)		Overall		P-value comparing CD in Cases (controls excluded)
	Case (N = 115)	Case (N = 191)	Control (N = 25)	Case (N = 306)	Control (N = 25)	
**DEMOGRAPHICS**						
**Age**						
Mean (SD)	57.2 (17.3)	62.6 (15.2)	50.0 (15.8)	60.5 (16.2)	50.0 (15.8)	0.00614
**Gender**						
Male	55 (47.8%)	96 (50.3%)	14 (56.0%)	151 (49.3%)	14 (56.0%)	
Female	60 (52.2%)	94 (49.2%)	11 (44.0%)	154 (50.3%)	11 (44.0%)	0.735
Missing	0 (0%)	1 (0.5%)	0 (0%)	1 (0.3%)	0 (0%)	
**Race**						
Black	43 (37.4%)	68 (35.6%)	13 (52.0%)	111 (36.3%)	13 (52.0%)	0.830
White	72.0 (62.6%)	120 (62.8%)	11.0 (44.0%)	192 (62.7%)	11.0 (44.0%)	
American Indian/Alaskan native	0 (0%)	1 (0.5%)	0 (0%)	1 (0.3%)	0 (0%)	
Asian	0 (0%)	0 (0%)	1 (4.0%)	0 (0%)	1 (4.0%)	
other	0 (0%)	1 (0.5%)	0 (0%)	1 (0.3%)	0 (0%)	
unknown	0 (0%)	1 (0.5%)	0 (0%)	1 (0.3%)	0 (0%)	
**Ethnicity**						
Hispanic	5 (4.3%)	9 (4.7%)	0 (0%)	14 (4.6%)	0 (0%)	0.425
unknown	1 (0.9%)	6 (3.1%)	0 (0%)	7 (2.3%)	0 (0%)	
Missing	0 (0%)	1 (0.5%)	1 (4.0%)	1 (0.3%)	1 (4.0%)	
**VITAL SIGNS at PRESENTATION**						
**Initial Systolic Blood pressure**						
Mean (SD)	125 (27.6)	135 (23.0)	129 (18.5)	132 (25.2)	129 (18.5)	0.00165
Missing	1 (0.9%)	0 (0%)	0 (0%)	1 (0.3%)	0 (0%)	
**Initial HR**						
Mean (SD)	111 (22.1)	101 (18.7)	95.7 (20.8)	104 (20.6)	95.7 (20.8)	<0.001
Missing	1 (0.9%)	0 (0%)	0 (0%)	1 (0.3%)	0 (0%)	
**Lowest Systolic BP (within 3 hrs)**						
Mean (SD)	110 (25.9)	125 (21.3)	119 (22.7)	119 (24.2)	119 (22.7)	<0.001

Missing	1 (0.9%)	0 (0%)	1 (4.0%)	1 (0.3%)	1 (4.0%)	
**Highest HR (within 3 hrs)**						
Mean (SD)	116 (20.6)	103 (18.3)	99.2 (19.7)	108 (20.2)	99.2 (19.7)	<0.001
**Initial Shock Index**						
Mean (SD)	0.915 (0.255)	0.764 (0.202)	0.748 (0.233)	0.820 (0.235)	0.748 (0.233)	<0.001
Missing	2 (1.7%)	0 (0%)	0 (0%)	2 (0.7%)	0 (0%)	
**Initial RR**						
Mean (SD)	21.2 (5.66)	20.2 (4.03)	18.3 (2.81)	20.6 (4.72)	18.3 (2.81)	0.108
Missing	1 (0.9%)	0 (0%)	0 (0%)	1 (0.3%)	0 (0%)	
**Highest RR**						
Mean (SD)	30.0 (7.88)	26.4 (15.6)	21.9 (5.44)	27.7 (13.4)	21.9 (5.44)	0.00759
**Initial O2 Saturation**						
Mean (SD)	93.2 (7.90)	95.7 (3.85)	96.6 (4.37)	94.8 (5.84)	96.6 (4.37)	0.00138
Missing	1 (0.9%)	0 (0%)	0 (0%)	1 (0.3%)	0 (0%)	
**Lowest O2 Saturation**						
Mean (SD)	88.8 (8.57)	91.5 (4.40)	94.5 (5.22)	90.5 (6.41)	94.5 (5.22)	0.002
Missing	1 (0.9%)	0 (0%)	0 (0%)	1 (0.3%)	0 (0%)	
**BMI**						
Mean (SD)	33.1 (8.36)	32.8 (9.29)	31.0 (9.41)	32.9 (8.94)	31.0 (9.41)	0.788
**Body Surface Area (BSA)**						
Mean (SD)	2.12 (0.325)	2.09 (0.333)	2.06 (0.347)	2.10 (0.330)	2.06 (0.347)	0.4
Missing	2 (1.7%)	7 (3.7%)	0 (0%)	9 (2.9%)	0 (0%)	
**First Temperature in ED (Fahrenheit degrees)**						
Mean (SD)	98.1 (0.777)	98.3 (0.791)	98.9 (1.46)	98.2 (0.790)	98.9 (1.46)	0.0416
Missing	5 (4.3%)	5 (2.6%)	0 (0%)	10 (3.3%)	0 (0%)	
**Transient hypotension prior to enrollment?**						
Yes	28 (24.3%)	7 (3.7%)	0 (0%)	35 (11.4%)	0 (0%)	<0.001
**Preceding episodes of syncope?**						
No	87 (75.7%)	162 (84.8%)	23 (92.0%)	249 (81.4%)	23 (92.0%)	0.0654
Yes	28 (24.3%)	29 (15.2%)	2 (8.0%)	57 (18.6%)	2 (8.0%)	

PE Risk FACTORS						
**Prior diagnosis of PE or DVT?**						
Yes	23 (20.0%)	46.0 (24.1%)	10 (40.0%)	69 (22.5%)	10 (40.0%)	0.492
**Recent hospitalization (in 3 weeks)?**						
No	93 (80.9%)	164 (85.9%)	17 (68.0%)	257 (84.0%)	17 (68.0%)	0.346
Yes	21 (18.3%)	26 (13.6%)	8 (32.0%)	47 (15.4%)	8 (32.0%)	
Missing	1 (0.9%)	1 (0.5%)	0 (0%)	2 (0.7%)	0 (0%)	
**Anticoagulation use?**						
Yes	7 (6.1%)	10 (5.2%)	3 (12.0%)	17 (5.6%)	3 (12.0%)	0.963
Missing	0 (0%)	1 (0.5%)	0 (0%)	1 (0.3%)	0 (0%)	
**Current or recent pregnancy (or miscarriage) within 6 weeks**						
Yes	4 (3.5%)	0 (0%)	0 (0%)	4 (1.3%)	0 (0%)	0.038
**Recent limb immobilization (current or within 3 weeks)**						
Yes	16 (13.9%)	10 (5.2%)	3 (12.0%)	26 (8.5%)	3 (12.0%)	0.0143
Missing	1 (0.9%)	0 (0%)	2. (8.0%)	1 (0.3%)	2 (8.0%)	
**Recent trauma (in the last 4–6 weeks)?**						
Yes	10.0 (8.7%)	13 (6.8%)	2 (8.0%)	23 (7.5%)	2 (8.0%)	0.686
Missing	1 (0.9%)	0 (0%)	0 (0%)	1 (0.3%)	0 (0%)	
**Surgery (requiring mechanical ventilation or epidural) within 6 weeks?**						
Yes	11 (9.6%)	19 (9.9%)	3 (12.0%)	30 (9.8%)	3 (12.0%)	1
Missing	1 (0.9%)	0 (0%)	2 (8.0%)	1 (0.3%)	2 (8.0%)	
**Family history of VTE?**						
Yes	7 (6.1%)	16 (8.4%)	1 (4.0%)	23 (7.5%)	1 (4.0%)	0.591
Missing	0 (0%)	2 (1.0%)	0 (0%)	2 (0.7%)	0 (0%)	
**Clotting disorders (protein, c, s, Factor V...)?**						
Yes	6 (5.2%)	4 (2.1%)	1 (4.0%)	10 (3.3%)	1 (4.0%)	0.241
Missing	1 (0.9%)	0 (0%)	0 (0%)	1 (0.3%)	0 (0%)	

**Hormone Replacement Therapy**						
Yes	12 (10.4%)	13 (6.8%)	3 (12.0%)	25 (8.2%)	3 (12.0%)	0.367
Missing	1 (0.9%)	2 (1.0%)	0 (0%)	3 (1.0%)	0 (0%)	
**Indwelling Vascular catheter? (e.g., portacath)**						
Yes	2 (1.7%)	13 (6.8%)	3 (12.0%)	15 (4.9%)	3 (12.0%)	0.0891
Missing	1 (0.9%)	0 (0%)	0 (0%)	1 (0.3%)	0 (0%)	
**Tobacco smoker**						
Yes	18 (15.7%)	41 (21.5%)	7 (28.0%)	59 (19.3%)	7 (28.0%)	0.272
Missing	0 (0%)	0 (0%)	1 (4.0%)	0 (0%)	1 (4.0%)	
**COMORBIDITIES**						
**Known pulmonary hypertension**						
Yes	36 (31.3%)	44 (23.0%)	0 (0%)	80 (26.1%)	0 (0%)	0.132
Missing	1 (0.9%)	0 (0%)	1 (4.0%)	1 (0.3%)	1 (4.0%)	
**Suspected/confirmed acute myocardial infarction**						
Yes	3 (2.6%)	4 (2.1%)	0 (0%)	7 (2.3%)	0 (0%)	1
**Suspected/confirmed sepsis?**						
Yes	3 (2.6%)	3 (1.6%)	1 (4.0%)	6 (2.0%)	1 (4.0%)	0.84
Missing	0 (0%)	1 (0.5%)	0 (0%)	1 (0.3%)	0 (0%)	
**Suspected/confirmed hypovolemia?**						
Yes	12 (10.4%)	11 (5.8%)	0 (0%)	23 (7.5%)	0 (0%)	0.211
Missing	0 (0%)	2 (1.0%)	0 (0%)	2 (0.7%)	0 (0%)	
**Suspected/confirmed unstable dysrhythmia?**						
Yes	5 (4.3%)	0 (0%)	0 (0%)	5 (1.6%)	0 (0%)	0.015
Missing	0 (0%)	1 (0.5%)	0 (0%)	1 (0.3%)	0 (0%)	
**Suspected/Confirmed severe LV dysfunction?**						
Yes	13 (11.3%)	4 (2.1%)	2 (8.0%)	17 (5.6%)	2 (8.0%)	0.00171
Missing	0 (0%)	1 (0.5%)	0 (0%)	1 (0.3%)	0 (0%)	
**Severe renal impairment? (Glomerular Filtration Rate < 30)**						
Yes	8 (7.0%)	6 (3.1%)	0 (0%)	14 (4.6%)	0 (0%)	0.206

**Chronic pulmonary disease**						
Yes	21 (18.3%)	38 (19.9%)	2 (8.0%)	59 (19.3%)	2 (8.0%)	0.84
**Severe Liver Impairment?**						
Yes	2 (1.7%)	0 (0%)	2 (8.0%)	2 (0.7%)	2 (8.0%)	0.273
**Rheumatologic disease (for example SLE, RA)**						
No	112 (97.4%)	185 (96.9%)	24.0 (96.0%)	297 (97.1%)	24.0 (96.0%)	0.717
Yes	2 (1.7%)	6 (3.1%)	1 (4.0%)	8 (2.6%)	1 (4.0%)	
Missing	1 (0.9%)	0 (0%)	0 (0%)	1 (0.3%)	0 (0%)	
**Diabetes with end-organ damage**						
Yes	8 (7.0%)	20 (10.5%)	0 (0%)	28 (9.2%)	0 (0%)	0.408
**Renal disease**						
Yes	14 (12.2%)	34 (17.8%)	0 (0%)	48 (15.7%)	0 (0%)	0.243
Missing	0 (0%)	1 (0.5%)	0 (0%)	1 (0.3%)	0 (0%)	
**Congestive Heart Failure**						
Yes	14 (12.2%)	11 (5.8%)	1 (4.0%)	25 (8.2%)	1 (4.0%)	0.073
Missing	1 (0.9%)	0 (0%)	1 (4.0%)	1 (0.3%)	1 (4.0%)	
**Mild liver disease**						
Yes	3 (2.6%)	6 (3.1%)	0 (0%)	9 (2.9%)	0 (0%)	1
Missing	1 (0.9%)	0 (0%)	0 (0%)	1 (0.3%)	0 (0%)	
**Dementia**						
Yes	5 (4.3%)	10 (5.2%)	0 (0%)	15 (4.9%)	0 (0%)	0.94
**Hemiplegia or paraplegia**						
Yes	4 (3.5%)	3 (1.6%)	1 (4.0%)	7 (2.3%)	1 (4.0%)	0.485
Missing	1 (0.9%)	0 (0%)	0 (0%)	1 (0.3%)	0 (0%)	
**Total Charlson Index**						
0	62 (53.9%)	93 (48.7%)	15 (60.0%)	155 (50.7%)	15 (60.0%)	0.609
1	18 (15.7%)	25 (13.1%)	1 (4.0%)	43 (14.1%)	1 (4.0%)	
2	10 (8.7%)	32 (16.8%)	3 (12.0%)	42 (13.7%)	3 (12.0%)	
3	6 (5.2%)	11 (5.8%)	1 (4.0%)	17 (5.6%)	1 (4.0%)	
4	6 (5.2%)	6 (3.1%)	2 (8.0%)	12 (3.9%)	2 (8.0%)	
5	3 (2.6%)	4 (2.1%)	0 (0%)	7 (2.3%)	0 (0%)	
6	3 (2.6%)	4 (2.1%)	2 (8.0%)	7 (2.3%)	2(8.0%)	
7	1 (0.9%)	7 (3.7%)	1 (4.0%)	8 (2.6%)	1 (4.0%)	

8	5.0 (4.3%)	5 (2.6%)	0 (0%)	10 (3.3%)	0 (0%)	
9	1 (0.9%)	2 (1.0%)	0 (0%)	3 (1.0%)	0 (0%)	
10	0 (0%)	1 (0.5%)	0 (0%)	1 (0.3%)	0 (0%)	
11	0 (0%)	1 (0.5%)	0 (0%)	1 (0.3%)	0 (0%)	
**Metastatic solid tumor**						
Yes	9 (7.8%)	19 (9.9%)	3 (12.0%)	28 (9.2%)	3 (12.0%)	0.665
Missing	0 (0%)	1 (0.5%)	0 (0%)	1 (0.3%)	0 (0%)	
**AIDS / HIV**						
Yes	0 (0%)	1 (0.5%)	0 (0%)	1 (0.3%)	0 (0%)	1
**Moderate or severe liver disease**						
Yes	1 (0.9%)	1 (0.5%)	2 (8.0%)	2 (0.7%)	2 (8.0%)	1
**Any malignancy**						
Yes	11 (9.6%)	24 (12.6%)	2 (8.0%)	35 (11.4%)	2 (8.0%)	0.539
Missing	3 (2.6%)	5 (2.6%)	0 (0%)	8 (2.6%)	0 (0%)	
**Known Hospice or End of Life Status at Presentation**						
No	112 (97.4%)	187 (97.9%)	23.0 (92.0%)	299 (97.7%)	23.0 (92.0%)	0.84
Yes	3 (2.6%)	3 (1.6%)	1 (4.0%)	6 (2.0%)	1 (4.0%)	
Missing	0 (0%)	1 (0.5%)	1 (4.0%)	1 (0.3%)	1 (4.0%)	
**Time between CT result time and Echo time**						
Mean (SD)	11.9 (15.3)	12.4 (10.6)	12.3 (8.33)	12.2 (12.5)	12.3 (8.33)	0.759
Missing	6 (5.2%)	3 (1.6%)	0 (0%)	9 (2.9%)	0 (0%)	
**Anticoagulant Initiated?**						
Yes	105 (91.3%)	190 (99.5%)	25 (100%)	295 (96.4%)	25 (100%)	<0.001
**Unfractionated heparin**						
Yes	85.0 (73.9%)	110 (57.6%)	13.0 (52.0%)	195 (63.7%)	13.0 (52.0%)	0.0059
**Low molecular weight heparin**						
Yes	21 (18.3%)	80 (41.9%)	8 (32.0%)	101 (33.0%)	8 (32.0%)	<0.001
**Vitamin K antagonist**						
Yes	0 (0%)	0 (0%)	0 (0%)	0 (0%)	0(0%)	<0.001
**Factor Xa inhibitor**						
Yes	0 (0%)	1 (0.5%)	3 (12.0%)	1 (0.3%)	3 (12.0%)	1

**Active/High Risk of Bleeding**						
Yes	12 (10.4%)	14 (7.3%)	3 (12.0%)	26 (8.5%)	3 (12.0%)	0.45
Missing	1 (0.9%)	0 (0%)	1 (4.0%)	1 (0.3%)	1 (4.0%)	
**Any cancer?**						
Yes	18 (15.7%)	39 (20.4%)	5 (20.0%)	57 (18.6%)	5 (20.0%)	0.376
**Gender**						
Male	55 (47.8%)	96 (50.3%)	14 (56.0%)	151 (49.3%)	14 (56.0%)	
Female	60 (52.2%)	94 (49.2%)	11 (44.0%)	154 (50.3%)	11 (44.0%)	0.735
Missing	0 (0%)	1 (0.5%)	0 (0%)	1 (0.3%)	0 (0%)	
**Clinical Deterioration at 30 Days**						
Clinical Deterioration at 30 Days	115 (100%)	28 (14.7%)	3 (12.0%)	143 (46.7%)	3 (12.0%)	<0.001
**Group Assignment**						
No RVD based on Biomarkers or CT	5 (4.3%)	8 (4.2%)	20 (80.0%)	13 (4.2%)	20 (80.0%)	1
RVD based on at least 1 Biomarker or CT	105 (91.3%)	179 (93.7%)	4 (16.0%)	284 (92.8%)	4 (16.0%)	
Missing	5 (4.3%)	4 (2.1%)	1 (4.0%)	9 (2.9%)	1 (4.0%)	
**CT RV:LV Ratio 1.0 or more**						
Ratio= 1.0 or more	90 (78.3%)	147 (77.0%)	0 (0%)	237 (77.5%)	0 (0%)	0.585
Ratio <1.0	22 (19.1%)	44 (23%)	25 (100%)	66 (21.6%)	25 (100%)	
Missing	3 (2.6%)	0 (0%)	0 (0%)	3 (1.0%)	0 (0%)	
**CLINICAL DETERIORATION EVENTS**						
**Death within 5 days?**						
Yes	3 (2.6%)	0 (0%)	0 (0%)	3 (1.0%)	0 (0%)	0.1
**Cardiac arrest within 5 days?**						
Yes	11 (9.6%)	1 (0.5%)	0 (0%)	12 (3.9%)	0 (0%)	<0.001
**Resp Failure within 5 days?**						
Yes	32 (27.8%)	0 (0%)	0 (0%)	32 (10.5%)	0 (0%)	<0.001
**Dysrhythmia within 5 days?**						
Yes	31 (27.0%)	0 (0%)	0 (0%)	31 (10.1%)	0 (0%)	<0.001
**Reperfusion Intervention within 5 days**						

Yes	66 (57.4%)	0 (0%)	0 (0%)	66 (21.6%)	0 (0%)	<0.001
**Hypotension Pressors within 5 days**						
Yes	20 (17.4%)	0 (0%)	0 (0%)	20 (6.5%)	0 (0%)	<0.001
**Hypotension fluid within 5 days**						
Yes	44 (38.3%)	0 (0%)	0 (0%)	44 (14.4%)	0 (0%)	<0.001
**Major bleeding within 5 days**						
Yes	11 (9.6%)	2 (1.0%)	1 (4.0%)	13 (4.2%)	1 (4.0%)	0.00102
**Hypoxia within 5 days**						
Yes	88 (76.5%)	102 (53.4%)	9 (36.0%)	190 (62.1%)	9 (36.0%)	<0.001
**Clinical Deterioration within 30 days**						
Yes	115 (100%)	28 (14.7%)	3 (12.0%)	143 (46.7%)	3 (12.0%)	<0.001
**Recurrence of VTE**						
Yes	4 (3.5%)	1 (0.5%)	0 (0%)	5 (1.6%)	0 (0%)	0.12
Missing	8 (7.0%)	7 (3.7%)	0 (0%)	15 (4.9%)	0 (0%)	
**Death within 30 days**						
Yes	9 (7.8%)	4 (2.1%)	1 (4.0%)	13 (4.2%)	1 (4.0%)	0.0328
Missing	4 (3.5%)	5 (2.6%)	0 (0%)	9 (2.9%)	0 (0%)	
**Major bleeding within 30 days**						
Yes	6 (5.2%)	3 (1.6%)	1 (4.0%)	9 (2.9%)	1 (4.0%)	0.132
Missing	7 (6.1%)	9 (4.7%)	0 (0%)	16 (5.2%)	0 (0%)	

Abbreviations: CD = clinical deterioration, SD = standard deviation, A4 = apical 4 chamber window, abnlRV = abnormal RV features, BP = blood pressure, CT= computed tomography, HR= heart rate, RR = respiratory rate, BMI = body mass index, ED = emergency department, LV = left ventricle, SLE = systemic lupus erythematosus, RA = rheumatoid arthritis, RV = right ventricle, RVD = right ventricle abnormality, PE = pulmonary embolism, DVT = deep venous thrombosis, VTE = venous thromboembolism
