# Supplementary material for: Intermediate-risk pulmonary embolism: echocardiography predictors of clinical deterioration
Source: Crit Care. 2022 Jun 4;26:160. doi: 10.1186/s13054-022-04030-z (PMC9166499; doi:10.1186/s13054-022-04030-z)
Supplement: Supplementary file 3 — Additional file 3: Table S2. Echocardiography metrics by secondary outcome. [file 13054_2022_4030_MOESM3_ESM.pdf]

Table S2: Echocardiography metrics by secondary outcome

	Clinical Deterioration at 30 Days		No Clinical Deterioration (CD)		Overall		P-value comparing CD in Cases (controls excluded)
	**Case (N = 143)**	**Control (N = 3)**	**Case (N = 163)**	**Control (N = 22)**	**Case (N = 306)**	**Control (N = 25)**	
**Right Ventricle basal width (cm)**							
Mean (SD)	4.40 (0.813)	4.23 (0.709)	4.21 (0.733)	3.75 (0.695)	4.30 (0.776)	3.81 (0.700)	0.0309
**Left Ventricle basal width (cm)**							
Mean (SD)	4.11 (0.797)	4.73 (0.404)	4.24 (0.716)	4.51 (0.595)	4.18 (0.757)	4.54 (0.573)	0.12
Missing	2.00 (1.4%)	0 (0%)	4.00 (2.5%)	0 (0%)	6.00 (2.0%)	0 (0%)	
**Right Ventricle mid width (cm)**							
Mean (SD)	3.69 (0.830)	3.30 (0.656)	3.45 (0.891)	3.18 (0.851)	3.56 (0.870)	3.20 (0.819)	0.0151
Missing	2.00 (1.4%)	0 (0%)	2.00 (1.2%)	0 (0%)	4.00 (1.3%)	0 (0%)	
**RV:LV basal width ratio**							
Mean (SD)	1.11 (0.296)	0.900 (0.173)	1.02 (0.236)	0.836 (0.118)	1.06 (0.270)	0.844 (0.123)	0.00232
Missing	2.00 (1.4%)	0 (0%)	4.00 (2.5%)	0 (0%)	6.00 (2.0%)	0 (0%)	
**Tricuspid Annular Planar Systolic Excursion (TAPSE) (cm)**							
Mean (SD)	1.58 (0.536)	2.13 (0.404)	1.82 (0.519)	2.05 (0.512)	1.71 (0.539)	2.06 (0.494)	<0.001
Missing	9.00 (6.3%)	0 (0%)	3.00 (1.8%)	0 (0%)	12.0 (3.9%)	0 (0%)	

**Estimated PA pressure (mmHg)**							
Mean (SD)	48.4 (16.5)	38.3 (20.0)	47.5 (18.2)	35.4 (11.8)	47.9 (17.4)	36.2 (13.4)	0.683
Missing	18.0 (12.6%)	0 (0%)	33.0 (20.2%)	14.0 (63.6%)	51.0 (16.7%)	14.0 (56.0%)	
**rvsp_cat**							
1	18.0 (12.6%)	0 (0%)	33.0 (20.2%)	14.0 (63.6%)	51.0 (16.7%)	14.0 (56.0%)	0.072
2	23.0 (16.1%)	1.00 (33.3%)	37.0 (22.7%)	5.00 (22.7%)	60.0 (19.6%)	6.00 (24.0%)	
3	40.0 (28.0%)	1.00 (33.3%)	35.0 (21.5%)	2.00 (9.1%)	75.0 (24.5%)	3.00 (12.0%)	
4	32.0 (22.4%)	0 (0%)	23.0 (14.1%)	1.00 (4.5%)	55.0 (18.0%)	1.00 (4.0%)	
5	30.0 (21.0%)	1.00 (33.3%)	35.0 (21.5%)	0 (0%)	65.0 (21.2%)	1.00 (4.0%)	
**RV free wall systolic excursion velocity S', cm/s**							
Mean (SD)	10.5 (3.50)	16.0 (2.00)	12.3 (13.4)	13.7 (3.09)	11.4 (10.1)	14.0 (3.04)	0.124
Missing	11.0 (7.7%)	0 (0%)	12.0 (7.4%)	1.00 (4.5%)	23.0 (7.5%)	1.00 (4.0%)	
**Initial BNP Level (pg/mL)**							
Mean (SD)	430 (630)	37.0 (35.0)	242 (363)	42.9 (57.5)	329 (512)	42.2 (54.8)	0.00251
Missing	7.00 (4.9%)	0 (0%)	5.00 (3.1%)	0 (0%)	12.0 (3.9%)	0 (0%)	
**Initial Troponin Level (ng/mL)**							
Mean (SD)	0.313 (0.829)	0 (0)	0.156 (0.310)	0.0124 (0.0144)	0.237 (0.635)	0.0105 (0.0139)	0.118

Median [Min, Max]	0.0800 [0, 6.34]	0 [0, 0]	0.0400 [0, 2.10]	0.0100 [0, 0.0400]	0.0600 [0, 6.34]	0 [0, 0.0400]	
Missing	64.0 (44.8%)	0 (0%)	88.0 (54.0%)	5.00 (22.7%)	152 (49.7%)	5.00 (20.0%)	
**Initial High Sensitivity troponin, ng/L**							
Mean (SD)	437 (1510)	6.00 (NA)	101 (160)	11.6 (5.13)	243 (1000)	11.1 (5.15)	0.0627
Median [Min, Max]	102 [5.00, 12600]	6.00 [6.00, 6.00]	29.0 [0, 751]	12.0 [6.00, 19.0]	49.0 [0, 12600]	11.0 [6.00, 19.0]	
Missing	70.0 (49.0%)	2.00 (66.7%)	64.0 (39.3%)	12.0 (54.5%)	134 (43.8%)	14.0 (56.0%)	
**Troponin elevation?**							
Elevated	102 (71.3%)	0 (0%)	89.0 (54.6%)	3.00 (13.6%)	191 (62.4%)	3.00 (12.0%)	0.00378
Missing	0 (0%)	0 (0%)	0 (0%)	1.00 (4.5%)	0 (0%)	1.00 (4.0%)	

Abbreviations: RV = right ventricle, LV = left ventricle, PA = RVSP = right ventricle systolic pressure, BNP = brain natriuretic peptide
