# Supplementary material for: Intermediate-risk pulmonary embolism: echocardiography predictors of clinical deterioration
Source: Crit Care. 2022 Jun 4;26:160. doi: 10.1186/s13054-022-04030-z (PMC9166499; doi:10.1186/s13054-022-04030-z)
Supplement: Supplementary file 4 — Additional file 4: Table S3. Reduced right ventricle assessment logistic regression model for 30-day clinical deterioration. [file 13054_2022_4030_MOESM4_ESM.pdf]

Table S3: Reduced right ventricle assessment logistic regression model for 30-day clinical deterioration

	**Clinical Deterioration within 30 days**		
Predictors	*Odds Ratios*	*Confidence Interval*	*p-value*
(Intercept)	1.57	0.61–4.12	0.354
Initial BNP Level (per 100 pg/mL)	1.08	1.02–1.15	**0.008**
RV:LV basal width ratio (per 10% point increase in ratio)	1.15	1.05–1.26	**0.004**
Tricuspid annular planar systolic excursion (TAPSE, cm)	0.56	0.33–0.91	**0.022**
Observations	331		
R2 Tjur	0.106		

Abbreviations: BNP = brain natriuretic peptide, RV:LV = right ventricle to left ventricle ratio
