# Supplementary material for: Intermediate-risk pulmonary embolism: echocardiography predictors of clinical deterioration
Source: Crit Care. 2022 Jun 4;26:160. doi: 10.1186/s13054-022-04030-z (PMC9166499; doi:10.1186/s13054-022-04030-z)
Supplement: Supplementary file 5 — Additional file 5: Table S4. Final logistic model for 30-day clinical deterioration. [file 13054_2022_4030_MOESM5_ESM.pdf]

Table S4: Final logistic model for 30-day clinical deterioration

	**Clinical Deterioration within 30 days**		
Predictors	*Odds Ratios*	*Confidence Interval*	*p-value*
(Intercept)	0.99	0.06–30.00	0.994
Initial BNP Level (per 100 pg/mL)	1.07	1.01–1.14	**0.041**
RV:LV basal width ratio (per 10% point increase in ratio)	1.11	1.00–1.23	0.055
Tricuspid annular planar systolic excursion (TAPSE, cm)	0.65	0.37–1.11	0.118
Highest heart rate (within 3 hrs)	1.02	1.01–1.03	**0.006**
Transient hypotension prior to enrollment	3.85	1.48–11.62	**0.009**
Severe renal impairment	3.93	0.98–19.97	0.067
Anticoagulant initiated	0.14	0.01–0.89	0.080
Observations	331		
R2 Tjur	0.193		

* Abbreviations: BNP = brain natriuretic peptide, RV:LV = right ventricle to left ventricle ratio
