# Supplementary material for: Intermediate-risk pulmonary embolism: echocardiography predictors of clinical deterioration
Source: Crit Care. 2022 Jun 4;26:160. doi: 10.1186/s13054-022-04030-z (PMC9166499; doi:10.1186/s13054-022-04030-z)
Supplement: Supplementary file 6 — Additional file 6: Table S5. Comparison of random forest and logistic regression on 5-day clinical deterioration over 500 random test-validation data splits (mean metrics with 95% coverage intervals*)**. [file 13054_2022_4030_MOESM6_ESM.pdf]

Table S5: Comparison of random forest and logistic regression on 5-day clinical deterioration over 500 random test-validation data splits (mean metrics with 95% coverage intervals*)**

Model	Mean AUC	Mean Sensitivity	Mean Specificity	Positive Predictive value	Negative Predictive Value	Mean Delong's P-value
Random Forest	0.78 (0.70, 0.85)	0.73 (0.57, 0.86)	0.69 (0.57, 0.81)	0.56 (0.47, 0.67)	0.83 (0.75, 0.90)	0.64 (0.21, 0.98)
Logistic Regression	0.80 (0.73, 0.87)	0.54 (0.37, 0.69)	0.87 (0.78, 0.95)	0.70 (0.58, 0.84)	0.78 (0.72, 0.84)	NA

* *Sensitivity, specificity, positive predictive value, and negative predictive value are based on predictions using the default classification prediction threshold of 0.50.*

** *95% coverage intervals represent the middle 95% of values observed over the 500 iterations.*

Abbreviations: AUC = area under the curve
