# Supplementary figures and images for: Intermediate-risk pulmonary embolism: echocardiography predictors of clinical deterioration
Source: Crit Care. 2022 Jun 4;26:160. doi: 10.1186/s13054-022-04030-z (PMC9166499; doi:10.1186/s13054-022-04030-z)

## Variable importance for predicting 30-day outcome events

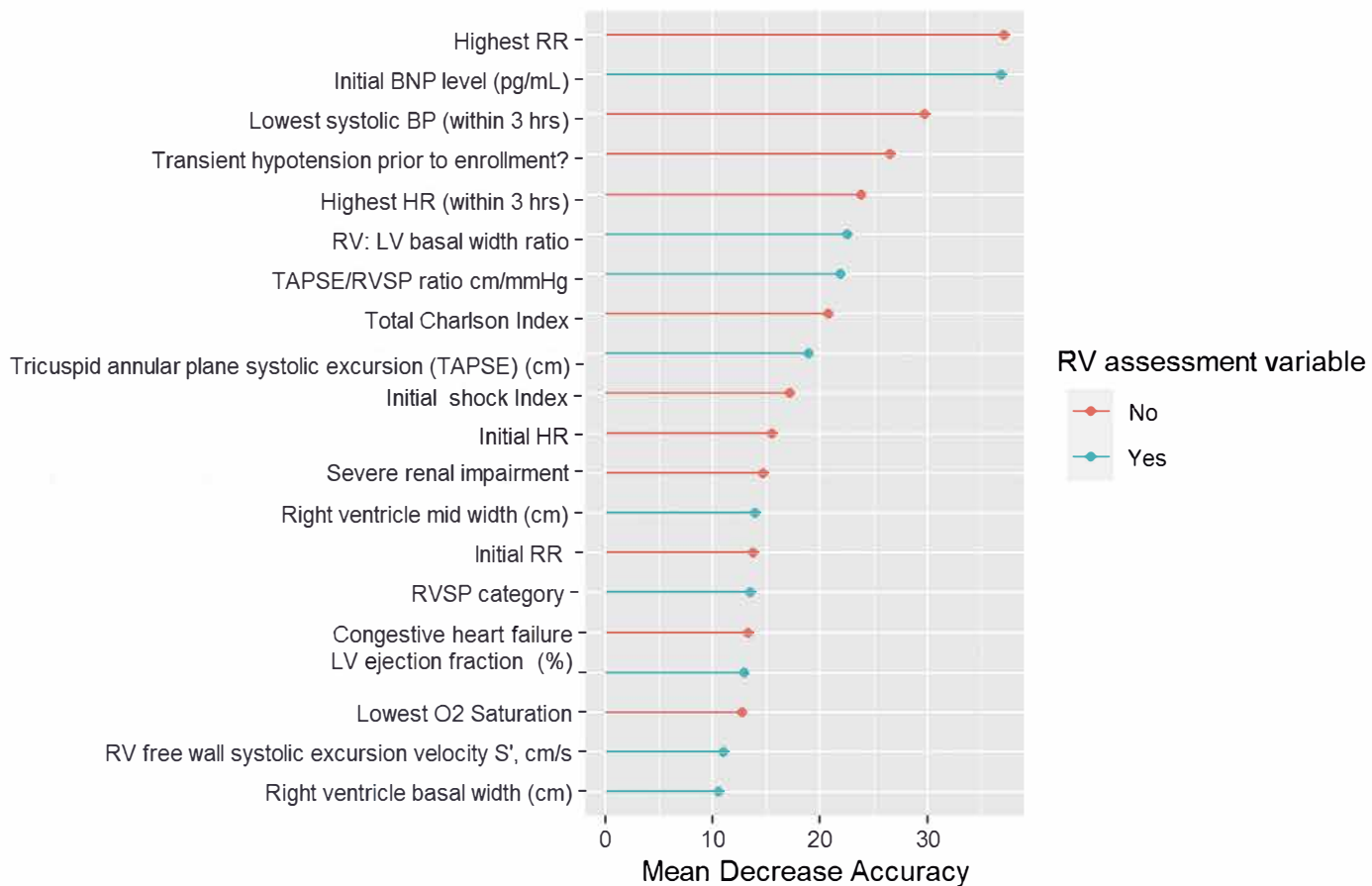

Supplement: Supplementary file 7 — Additional file 7: Fig. S1. Variable importance plot for random forest prognostic model for the secondary outcome. Abbreviations: A4 = apical 4-chamber window, BMI = body mass index, BP = blood pressure, CT = computed tomography, HR = heart rate, LV = left ventricle, Min = minimum, Max = maximum, RR = respiratory rate, RV = right ventricle, RVD = right ventricle abnormality, RVSP cat = right ventricle systolic pressure category, SD = standard deviation, TAPSE = tricuspid annular planar systolic excursion. [file 13054_2022_4030_MOESM7_ESM.pdf]
